# SARS‐CoV‐2 indoor environment contamination with epidemiological and experimental investigations

**DOI:** 10.1111/ina.13118

**Published:** 2022-10-24

**Authors:** Lotta‐Maria A. H. Oksanen, Jenni Virtanen, Enni Sanmark, Noora Rantanen, Vinaya Venkat, Svetlana Sofieva, Kirsi Aaltonen, Ilkka Kivistö, Julija Svirskaite, Aurora Díaz Pérez, Joel Kuula, Lev Levanov, Antti‐Pekka Hyvärinen, Leena Maunula, Nina S. Atanasova, Sirpa Laitinen, Veli‐Jukka Anttila, Lasse Lehtonen, Maija Lappalainen, Ahmed Geneid, Tarja Sironen

**Affiliations:** ^1^ Faculty of Medicine University of Helsinki Helsinki Finland; ^2^ Department of Otorhinolaryngology and Phoniatrics – Head and Neck Surgery Helsinki University Hospital Helsinki Finland; ^3^ Faculty of Veterinary Medicine University of Helsinki Helsinki Finland; ^4^ Faculty of Biological and Environmental Sciences University of Helsinki Helsinki Finland; ^5^ Finnish Meteorological Institute Helsinki Finland; ^6^ Finnish Institute of Occupational Health Kuopio Finland; ^7^ HUS Inflammation Center Helsinki University Hospital Helsinki Finland; ^8^ HUS Diagnostic Center, HUSLAB Helsinki University Hospital Helsinki Finland

**Keywords:** air sample, infection control, neutralizing antibody response, SARS‐CoV‐2, surface sample

## Abstract

SARS‐CoV‐2 has been detected both in air and on surfaces, but questions remain about the patient‐specific and environmental factors affecting virus transmission. Additionally, more detailed information on viral sampling of the air is needed. This prospective cohort study (*N* = 56) presents results from 258 air and 252 surface samples from the surroundings of 23 hospitalized and eight home‐treated COVID‐19 index patients between July 2020 and March 2021 and compares the results between the measured environments and patient factors. Additionally, epidemiological and experimental investigations were performed. The proportions of qRT‐PCR‐positive air (10.7% hospital/17.6% homes) and surface samples (8.8%/12.9%) showed statistical similarity in hospital and homes. Significant SARS‐CoV‐2 air contamination was observed in a large (655.25 m^3^) mechanically ventilated (1.67 air changes per hour, 32.4–421 L/s/patient) patient hall even with only two patients present. All positive air samples were obtained in the absence of aerosol‐generating procedures. In four cases, positive environmental samples were detected after the patients had developed a neutralizing IgG response. SARS‐CoV‐2 RNA was detected in the following particle sizes: 0.65–4.7 μm, 7.0**–**12.0 μm, >10 μm, and <100 μm. Appropriate infection control against airborne and surface transmission routes is needed in both environments, even after antibody production has begun.

**Practical implications**

- The finding of SARS‐CoV‐2 RNA from the air in the absence of aerosol generating procedures (AGP) and in the absence of respiratory symptoms emphasizes the use of respiratory protection and airborne precautions also in situations where AGPs are not performed and regardless of the patients’ symptoms.
- Families that used respiratory protection were able to prevent further infections.
- Air and surface contamination was detected in both homes and hospital even though the day from the start of the symptoms was later in hospital measurements. This may follow from more severe disease and increased viral loads which were associated with older age. Infection control measures should be used in both environments to prevent further infections.

## 1 INTRODUCTION

Increasing scientific evidence indicates the dominance of short‐ and long‐range airborne transmission of SARS‐CoV‐2.^1^, ^2^, ^3^, ^4^, ^5^, ^6^ The observed transmission risks have been higher indoors than outdoors,^7^ and discussion on precautions for hospital and home environments has been intense. In a study that aerosolized SARS‐CoV‐2 under laboratory conditions, aerosols' infectivity was retained for up to 16 h,^8^ while another study estimated the half‐life in aerosols to be approximately 1.1–1.2 h (95% CI 0.64–2.64).^9^ Outside of the laboratory, signs of viable SARS‐CoV‐2 in the air have been detected,^10^, ^11^, ^12^ and the virus has also been cultured from exhaled air.^13^ A direct link between SARS‐CoV‐2 viral load, emission, and airborne concentration was recently demonstrated by Buonanno et al.^14^ A few studies have detected SARS‐CoV‐2 RNA in the air at home‐environment.^15^, ^16^ In hospitals, PCR‐based studies have found SARS‐CoV‐2 RNA in room air,^17^, ^18^, ^19^, ^20^, ^21^, ^22^, ^23^, ^24^, ^25^ as well as from air conditioning filters located over 50 m from the patient room.^26^ Even though previous studies have mainly used long collection times or high flow rates, challenge is that only a proportion of the air present in a room can be analyzed. Additionally, indoor turbulence highly affects local concentrations.^27^, ^28^ Thus, questions remain about the risk of infection during shorter meetings or in rooms with a larger air space, and whether the findings would be similar in the home environment. As environmental sampling is highly demanding and sample sizes rather small, more patient data are also needed to draw further conclusions in future systematic reviews.

According to laboratory studies, the stability of SARS‐CoV‐2 on surfaces varies depending on the surface type and environmental conditions.^9^, ^29^, ^30^, ^31^, ^32^ However, its ability to sustain infectivity on surfaces outside laboratory conditions is largely unknown.^33^ SARS‐CoV‐2 RNA has been found, for example, on high‐touch surfaces, floors, and toilets,^18^, ^19^, ^34^, ^35^ and there are a few possibly positive culture findings of SARS‐CoV‐2 from the surfaces.^36^, ^37^, ^38^ The effect of age and neutralizing antibodies (NAbs) on the spread of SARS‐CoV‐2 has been speculated,^39^, ^40^, ^41^ but there is a lack of clear evidence for the role of patient‐related factors.

This study sought to increase knowledge of SARS‐CoV‐2 transmission in different environments by analyzing air, surface, and patient samples from a COVID‐19 cohort ward in Helsinki University Hospital (HUS), Finland, and from patients' homes. The aims were to determine whether SARS‐CoV‐2 RNA or viable virus could be found in the home and hospital environments, and which patient‐ and environment‐related factors affect the risk of environmental contamination. A team consisting of researchers from HUS, the University of Helsinki, the Finnish Meteorological Institute, and the Finnish Institute of Occupational Health was established to enable a multidisciplinary approach to the above research questions.

## 2 METHODS

### 2.1 Index patients and safety measures

**FIGURE 1:**
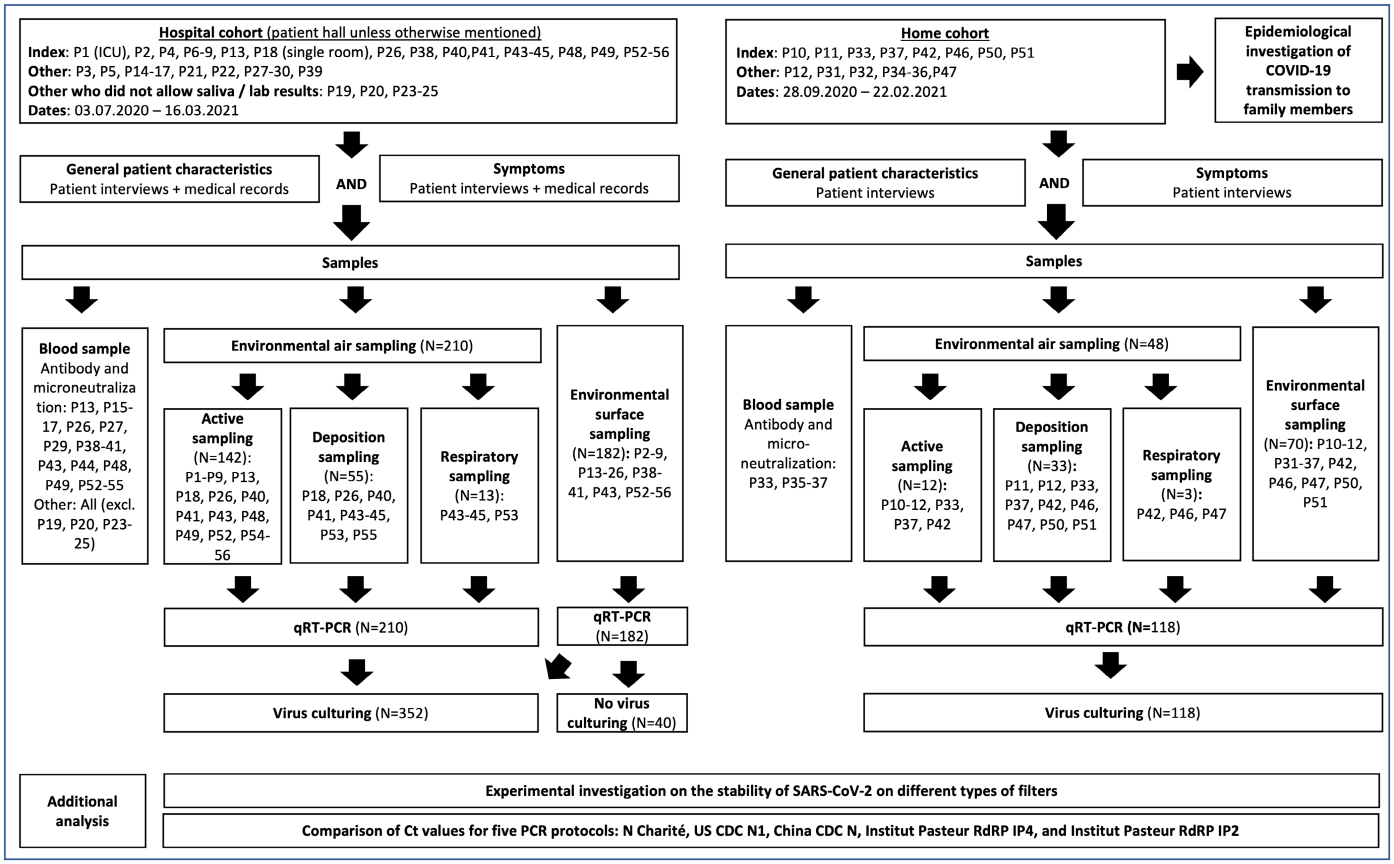
Patient inclusion and sampling process and additional analysis performed in the study

Patients were voluntary participants with a qRT‐PCR‐confirmed symptomatic COVID‐19 infection between 1.7.2020 and 16.3.2021. None of the participants had been vaccinated. As infectivity has been observed to be highest in early disease, the patient with the most recent onset of symptoms was selected as the index patient,^42^ except for collection 13, where all the patients in the room had been symptomatic for over 10 days and the patient with the freshest positive PCR result (P26) was selected (Table S1). Environmental measurements were performed in the vicinity of the index patients. Saliva samples were also collected from other patients who were in the ward at the same time and who agreed to the study in 4 collections (named as “other”). Family members of the home patients were examined for infection and seroconversion. Environmental sampling was performed twice with patients P2 and P3, and P2 was considered as an index due to the more recent start of the symptoms; however, some personal items from both were sampled. The sampling process is presented in a flow chart in Figure 1.

All research personnel conducting the sampling followed aerosol safety protocols and precautions and no infections were detected. All procedures that involved human participants, including environmental sampling, were conducted in accordance with the ethical standards of the institutional or national research committee and the 1964 Declaration of Helsinki and its later amendments or comparable ethical standards. The Ethics Committee of Helsinki University Hospital approved the study protocol (HUS/1701/2020). All respondents provided written informed consent prior to their participation.

### 2.2 Infection prevention protocols in the hospital

The infection prevention and control protocols on the COVID ward included hand hygiene, universal masking for staff (FFP2/3 for ICU and surgical masks for the COVID ward), guidance on social distancing (2 m), and personal protecting equipment (PPE) following droplet precautions. The patients did not use face masks. The ward and ICU were cleaned twice a day between 9 to 10 am and 4 to 5 pm. The sample collections were done between cleaning around 11 am to 9 pm and thus reflect quite reliably patients' infection status of the collection day. The specific cleaning protocol is presented in the supplement.

### 2.3 Cell lines

Vero E6 cells (VE6) and their TMPRSS2‐expressing clone VE6‐TMPRSS2‐10 (VE6T)^43^ were grown as previously described.^44^ To inhibit fungal growth, 0.205 μg/ml of amphotericin B (Fungizone, Thermo Scientific) was added to the medium of the cells that were taken to the hospital for aerosol collections. The used VE6 cell line is originally from ATCC (American type Culture Collection^45^), and the VE6T cell line has been modified from the original line according to the previous study.^43^

### 2.4 Sampling protocols for air sampling

Seven different air collection methods were used. Details of the collections and samples are presented in Table S3. Sampling times and air volumes varied between different sampling methods depending on the expected optimal collection time for each device according to the manufacturer and previous studies, and the knowledge gathered during the study. A Dekati PM10 cascade impactor (20 L/min air flow, model PMS‐420) with three stages (>10.0 μm, 2.5–10 μm, and 1.0–2.5 μm), including a backup filter for particles <1 μm, was used in 11 collections. The three impaction stages were fitted with 25‐mm‐diameter cellulose acetate membrane filters (CA filter, GE Healthcare Life Sciences) and the backup plate with a 40‐mm CA filter. Analyzing the three stages and backup filter, particle distribution according to aerodynamic size (PM10, PM2.5, and PM1) can be ascertained. The collector was placed within 1–2 m from the patient, and particles were collected for 2–4 h. After sampling, filters were immediately placed in 2 ml (25‐mm filter) or 3 ml (40‐mm filter) of minimal essential Eagle's medium (MEM, Sigma‐Aldrich).

The BioSpot 300p bioaerosol sampler prototype (Aerosol Devices Inc.) has a flow rate of 8 L/min and a mechanism that allows water to condense on aerosol particles from as small as 5–10 nm to 20 μm in diameter and minimize the stress when the sample is impacted onto the surface with the collection medium. To increase the sample collection rate, the biosampler is equipped with eight wicking tubes fitted with three nozzle jets to secure gentle transfer of the sample. This sampler was used in 8 collections for 1.5–4 h within a distance of 1–2.5 m from the patient, and the sample was collected in 1–2 ml of MEM.

As a more portable solution for personal area air sampling, a standard 25‐mm gelatin (Sartorius Stedim Biotech) or mixed cellulose ester (MCE) filter equipped in the Button sampler with a Gilian 5000 air sampling pump, 4 L/min air flow, and a porous curved surface inlet was used in 9 collections. The Button sampler collects particles smaller than 100 μm.^46^ The stability of SARS‐COV‐2 on two filter materials was compared under laboratory conditions to select the more optimal filter type and to optimize the collection time (details in Supplementary Material). Samples were collected for 10–30 min from patients' breathing area. Depending on the health status, a conversation was prompted to increase the output of aerosols. The collection filter was removed into 3 ml of MEM immediately after collection ended.

Three Andersen cascade impactors (400 W pump and 28.3 L/min flow rate) were used simultaneously in six collections. The impactors consist of six stages with size cut points of (1) >7 μm, (2) 4.7–7.0 μm, (3) 3.3–4.7 μm, (4) 2.1–3.3 μm, (5) 1.1–2.1 μm, and (6) 0.65–1.1 μm. An additional inlet was used during measurements limiting the upper limit of the particle size to 12 μm. To ensure the correct volume flow rate, each Andersen impactor was fitted with a TSI flow meter. Samples were collected using Petri dishes (94/16 MM) with 15 ml of cell medium for 10, 20, and 30 min. The medium was transferred onto VE6T cells grown on 100/20 MM cell culture dishes either immediately after collection in the hospital (collections 24, 25, 27, and 29) or later in the laboratory (collection 31).

To evaluate the real‐time particle number concentration during the hospital collections and to gather additional air samples, a Dekati eFilter was used in two collections. The eFilter monitors changes in real‐time particle concentration by utilizing a small diffusion charger powered by an inner chargeable battery. The charge changes were automatically translated into a signal, which was recorded on a data card. When postprocessing the data, the raw charge signal was further converted to represent particle number concentrations using a conversion factor (411 cm^−3^ fA^−1^) provided by the manufacturer. A count median diameter (CMD) of 60 nm and a geometric standard deviation (GSD) of 1.5 were assumed.^47^, ^48^ In addition, the eFilter simultaneously collected samples on a 47‐mm gelatin filter using an external pump. After sample collection, the gelatin filter was transferred into 6 ml of MEM. The eFilter was fitted with the same EPA‐designed inlets as the Andersen cascade impactors. The particle size cut point of the inlets was approximately 12 μm, with an air volume flow rate of 28.3 L/min. The duration of sample collection was 30 min at a similar distance from the patient as with Andersen's cascade impactors.

Passive air samples were collected either directly on VE6 cells (2 collections) or VE6T cells (9 collections) grown on 100/20 MM (collection 22) or 35/10 MM (other collections) cell culture dishes or on empty 35/10 MM Petri dishes containing 1 ml of growth medium (10 collections). Open dishes were positioned at different proximities from the patient for 30–60 min, and the patient was encouraged to perform an aerosol‐producing activity such as talking. The ability of SARS‐CoV‐2 to infect cells at room temperature was confirmed, and major differences in the culture sensitivity of these two collection methods were excluded under laboratory conditions (see Supplementary Methods for details).

Living cells were transported to the laboratory in a warm environment with heat accumulators warmed to 37°C. One plate was used as a negative control to ensure that the cells survived the transport. Other samples were transported with cold accumulators and handled during the same or next day.

### 2.5 Sampling protocols for surface sampling

Altogether, 252 surface samples in 26 collections for qRT‐PCR testing were taken from surfaces in possible direct or indirect contact with the patient (Table S3) with pre‐wetted Dacron swabs (Copan, 25 collections), a nitrile glove (1 collection), gauze (1 collection), or by pipetting the sampling liquid up and down on the surface a few times and transferring it into a sampling tube (3 collections). Swabs were placed into 1 ml of PBS. In 22 collections (212 samples), an additional sample was taken for virus culture, which was placed in 250 μl or 1 ml of MEM. Samples for qRT‐PCR and culturing were taken immediately next to each other. Surfaces were divided into four surface groups (high‐touch surfaces, low‐touch surfaces, toilet surfaces, and other surfaces) for statistical analyses.

### 2.6 Other sampling protocols

Saliva samples were taken from 26 index patients either with a Dacron swab (collections 5–9) or by spitting into a Falcon tube (from collection 10 onwards). Ten additional saliva samples were collected from other patients from the ward in four collections and from seven healthy family members of home‐treated patients. If possible, patients were asked to rinse their mouth before sampling. In collection 23, the index patient and a healthy family member also took follow‐up saliva samples until 12 days from the start of the patient's symptoms. In collection 26, follow‐up saliva samples were taken from patients until Days 14–17 from the start of symptoms.

Nasopharyngeal samples from consenting patients were taken and sent to HUSLAB for a fresh diagnostic PCR.^49^, ^50^ Serum samples from consenting patients were taken within a day from sampling and tested for SARS‐CoV‐2 IgG antibodies with two different tests.^51^ Serum samples (dilutions 1:10 to 1:640) were studied with the microneutralization assay.^52^ Blood lymphocyte and eosinophil counts, and plasma CRP from consenting patients were measured within a day of sampling, and plasma ferritin, ALP, ALT, D‐dimer, and fibrinogen levels within 3 days. The respiration rate and SpO2 levels were measured during the same day (Table S1).

Since the first cases caused by variants of concern (VoC) were detected in Finland at the end of December 2020, they were determined from all patients as a part of routine diagnostics. This information was used to compare the results between VoC strains (mainly alpha in Finland) and non‐VoC strains. Virus strains of collections 1–22 (P1–P45) were considered as non‐VoC, as they were collected before the first cases were reported in Finland.

### 2.7 RNA extraction and PCR protocols for air, saliva, and culture medium samples

Trizol (Invitrogen) was used to extract RNA from all saliva samples and from air and culture medium samples of collections 1–23 according to the manufacturers' instructions. A 200‐μl sample was added to 800 μl of Trizol reagent, and a resuspension volume of 50 μl was used. RNA was extracted from air and culture medium samples of collections 24 onwards with a QIAcube HT system and QIAamp 96 Virus QIAcube HT kit (QIAgen) using off‐board lysis.

All samples were tested with two different qRT‐PCRs, N Charité,^53^ and N1 US CDC,^54^ using TaqMan Fast Virus 1‐Step Master Mix (ThermoFisher), a 20‐μl reaction volume, 45 cycles/run, and fast cycling mode (annealing temperatures 55°C (N1 US CDC) and 58°C (N Charité)). The primer and probe concentrations of N Charité were according to the original publication,^53^ and those of N1 US CDC were 500 nM of both primers and 125 nM of probe (Table S7). qRT‐PCRs were performed using a Stratagene Mx3005P instrument (Agilent Technologies) with a Ct cutoff value of 0.04. The results were considered positive if both qRT‐PCRs were positive with a Ct value under 40 or if one qRT‐PCR was positive with a Ct value under 38. Comparable cutoff limits have been used in previous studies.^18^, ^55^, ^56^ Samples with Ct values over 38 in one qRT‐PCR and no Ct with the other one were treated as negative, even though the possibility of them being very weak positives could not be excluded. RNA extracted from the Fin/20 strain^52^ culture was used as a positive control and nuclease‐free water as a negative control. Limit of detection in different laboratories has been around 5 copies/reaction for N1 US CDC and up to 50 copies/reaction for N Charité.^57^

The N gene transcript for qRT‐PCR was prepared as follows: The target region (352–712, 360 bp) was amplified from SARS‐CoV‐2 RNA, Wuhan strain, and cloned into pGEM‐T cloning vector (Promega) under control of the SP6 promoter. The presence of the insert was verified by sequencing and restriction enzyme analysis. After linearization of the plasmid by digestion with AscI (Thermo Fisher Scientific), RNA was generated using the RiboMAX™ Large Scale RNA production system with SP6 polymerase (Promega) according to the manufacturer's instructions. The transcribed RNA was then treated with DNAse I and purified with the RNeasy Mini Kit (QIAGEN). Finally, RNA was quantified by spectrophotometry, and the RNA copy number was calculated based on its concentration, length, and molecular weight. qRT‐PCR was performed with N Charité PCR by including a transcript dilution series from 10 to 10^9^ copies/reaction in triplicate and samples in duplicate. Copy numbers as copies/ml of saliva were calculated by tracing back from copies/reaction reported by MxPro software (standard curve equation: *Y* = −3.570 × LOG[*X*] + 43.98, RSq = 0.984). Quantitating air samples in a similar way was unsuccessful due to weak positive samples and repeated freeze‐and‐thaw cycles and copy numbers for those were estimated based on the Ct values from initial qRT‐PCR and above equation from a different run but the same protocol.

### 2.8 RNA extraction and PCR protocols for surface samples

RNA was extracted with the NucliSENS miniMAG kit (Biomerieux). Process control virus (mengovirus) was added to at least half of the samples. Tubes containing PBS and swabs were mixed by vortexing, and swabs were moved to 1 ml of high pH tris‐glycine‐beef extract buffer (TGBE, pH 9.5). The tubes were vortexed again and agitated at 250 rpm for 5 min in an orbital shaker (IKAKS 2060 basic, Patterson Scientific), and the swabs were moved into a tube with 4 ml of lysis buffer, vortexed and agitated at 250 rpm for 10 min. PBS, TGBE, and lysis buffer were then combined, vortexed, and incubated for 10 min. PBS without process control virus was included as a negative control and PBS with process control virus as a positive control. The rest of the extraction was carried out according to the NucliSENS miniMAG kit instructions. The samples were further treated with the OneStep PCR Inhibitor Removal Kit (Zymo Research) according to the manufacturer's instructions.

Samples were tested for SARS‐CoV‐2 with modified versions of N Charité^53^ and N1 US CDC qRT‐PCRs^54^ and for process control virus.^58^ The RT‐qRT‐PCR was carried out using a QuantiTect Probe RT‐PCR kit (Qiagen). Reaction mixes included 10 μl of 2X QuantiTect Probe RT‐PCR Master Mix, 0.2 μl of QuantiTect RT mix, 0.6 μM of forward and 0.8 μM of reverse primer, 0.2 μM of probe for N Charité qRT‐PCR primers, and 5 μl of RNA template, and the volume was adjusted to 20 μl with water. For US CDC qRT‐PCR, final concentrations of 0.5 μM for both primers and 0.2 μM for the probe were used. For mengovirus qRT‐PCR, 1 μM of both primers and 0.2 μM of probe were used. N Charité and N1 US CDC runs included one 10^−4^ dilution of SARS‐CoV‐2 RNA extracted from cell‐grown virus as a standard positive control and one or two blanks as a standard negative control, and the reactions were performed in duplicate whenever the sample amount was sufficient. A Rotor Gene 3000 (Qiagen) real‐time PCR cycler was used. The cycling conditions were reverse transcription for 30 min at 53°C, a denaturation step at 95°C for 15 min, followed by 45 cycles of amplification/denaturation at 95°C for 15 s, annealing at 58°C for 45 s, and extension at 72°C for 45 s. The results were analyzed with the thermocycler software Rotor‐Gene 6.0.31 (Qiagen) using similar criteria as with other samples described above.

### 2.9 Culturing protocols

Samples were initially cultured in VE6 cells (collections 1–18), which were changed to VE6T cells after reports of these being more sensitive (collections 19–31).^47^ Air samples that were collected directly on cells were cultured as such, and the rest of the air and surface samples and 75 μl of saliva were used for culturing in 6‐well plates. Medium was added to the final volume of 3 ml (saliva) or 2 ml (other samples). E‐filter samples were cultured in two wells (3 ml/well). Samples were cultured at 37°C for 10–14 days and checked for cytopathic effect (CPE). A 200‐μl sample of culture medium was taken from those samples that had unclear results based on microscopic observation or possible CPE and tested with N Charité qRT‐PCR. Culturing was considered positive if CPE was detected and the Ct value of qRT‐PCR performed from the culture media was under 20. If Ct value was higher, it was judged to be caused by original (possibly noninfectious) virus in the sample instead of virus growth. All virus culturing was performed in a BSL3 laboratory. Optimization of the culturing protocols is described in more detail in the Supplementary Material.

### 2.10 Statistical tests and design

Statistical tests were carried out with SPSS IBM Statistics version 27. When comparing means between two independent groups, data were first tested for normality with the Shapiro–Wilk test before testing them either with the independent‐samples *t*‐test or a non‐parametric test (independent‐samples Mann–Whitney *U*‐test for two groups and independent‐samples Kruskal–Wallis test for more than two groups). For categorical data, the Fisher–Freeman–Halton exact test was used. Air and surface results of collections were compared with McNemar's test. Spearman's rank correlation coefficient was used for correlation testing. Mean values and standard deviations (normally distributed data), medians and interquartile ranges (non‐normally distributed data), or percentages (categorical data) of compared subgroups, test statistics, *p*‐values, and effect sizes (Cohen's d for the *t*‐test and *z*/$\sqrt{N}$ for the Mann–Whitney *U*‐test) are reported in Table S2. *p*‐values below 0.05 were considered statistically significant. Air or surface collections were considered positive if at least one of the samples from the collection was qRT‐PCR positive. Individual data points that were added to the boxplot figures were jittered in all dimensions using a uniform distribution. Figures were created either with Adobe Illustrator 2020 version 24.0.2^63^ or SPSS IBM Statistics version 27.^59^ With labeling and design, Microsoft PowerPoint for Microsoft 365 MSO version 2201^60^ and Microsoft Paint version 20H2^61^ were used.

## 3 RESULTS

### 3.1 Patient characteristics and collection surroundings

**FIGURE 2:**
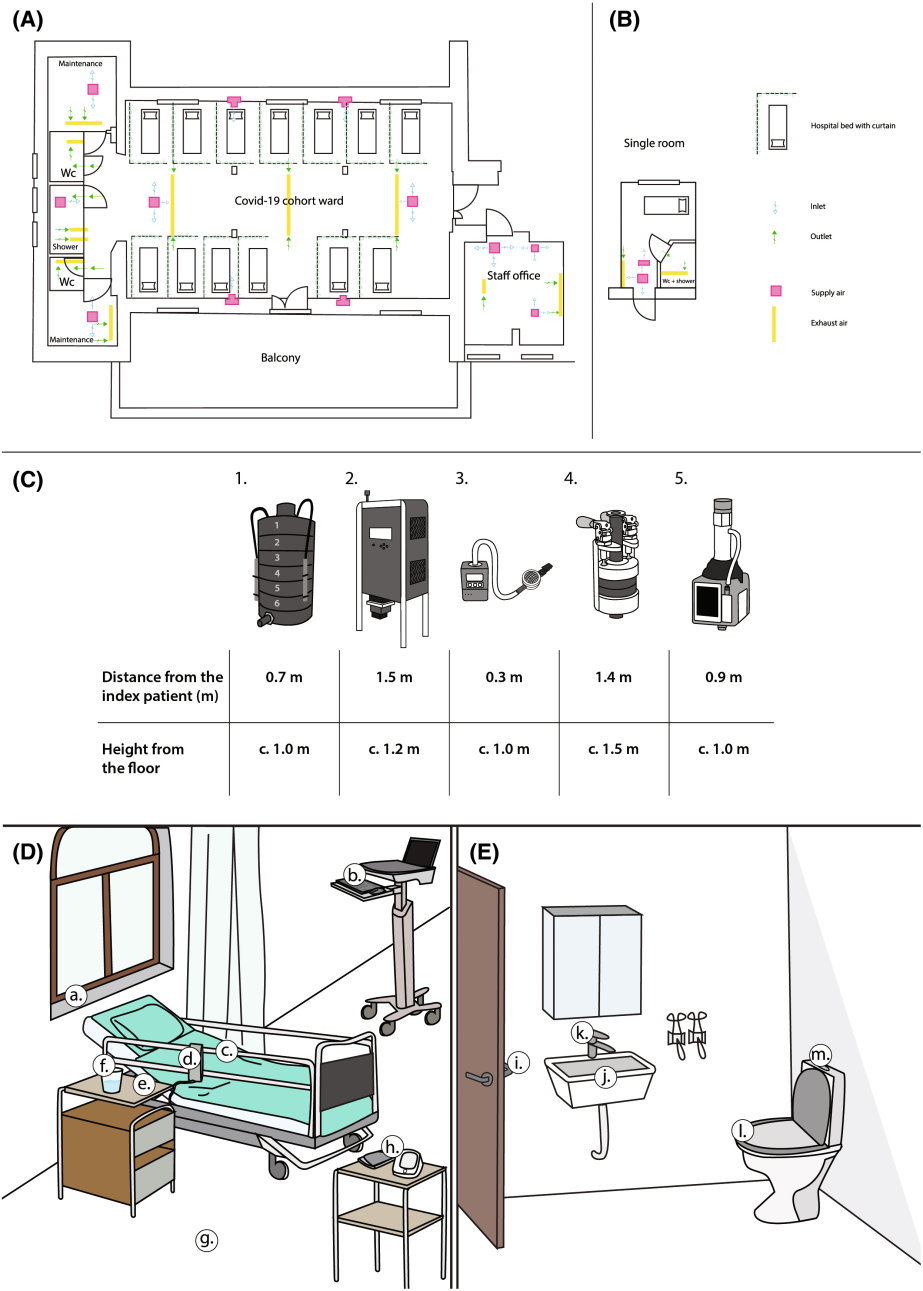
Sampling set up. (A) Layout of the COVID‐19 cohort ward. (B) Layout of the single‐patient room. (C) Locations of the sampling spots for the active air samplers around the patient (1: Andersen, 2: BioSpot, 3: Button, 4: Dekati, and 5: eFilter). (D) Example locations of the surface sampling spots and deposition sample spots around the patient, a: window sill, b: computer, c: bed rail, d: bed remote, e: table, f: drinking glass, g: floor, h: hospital equipment. (E) Location of the surface sampling spots in the toilet, i: door handle, j: toilet bowl, k: tap, l: toilet seat, m: toilet flush button. The figure was created with Adobe Illustrator 2020 (24.0.2)^63^

We performed 23 sample collections in HUS and 7 collections in patients' homes in the Uusimaa region, Finland, between July 2020 and March 2021. During the collection period, the COVID‐19 incidence in the Uusimaa area was 1–180 cases/100 000 inhabitants/7 days (average 61 cases/100 000 inhabitants per week) and the test positivity rate varied between 0.1% ‐ 4.5%.^62^ Collections included 56 patients of which 31 were index patients (1–2 per collection, see Methods for details), 21 of whom were treated on a COVID‐19 cohort ward in a large patient hall, one in a single‐patient room, one in the intensive care unit (ICU), and eight patients treated in their homes (Figure 2).

The cohort ward had an air volume of 655.25 m^3^ (height 4.5 m) and was provided with clean 100% outdoor supply air of 421 L/s generating 1.67 air changes per hour (ACH) and 32.4–421 L/s/patient depending on the number of patients. The mechanical ventilation type in the ward was displacement ventilation. The amount of supply air was increased before 16.3.2021 to further prevent staff infections. This change generated a higher 2.99 ACH for the last measurement (patient 56, P56). The single room in the ward had displacement ventilation with 100% outdoor supply air generating 4.7 ACH. The ICU room had a laminar inlet area in the middle of the room generating 20.52 overall ACH, and an outlet on the side of the room. All the air was filtered with HEPA13 before entering the room. The measured patient located partly under the laminar area. All measured home environments had automated mechanical ventilation, which is normal in Finland. The measured rooms were circa 15–30 m^2^ in area with a normal room height of 2.5 m. We were not able to measure the exact ACH at homes, but it is expected that they followed building regulations which recommend 0.5 ACH for homes. Patient characteristics, including symptoms and laboratory results, are summarized in Table 1 (see Tables S1 and S2 for details of the patients and statistical tests used throughout the manuscript).

**TABLE 1:** Characteristics of hospital‐treated and home‐treated index patients and statistical differences between the two groups

	Hospital (*N* = 23)	Home^a^ (*N* = 8)	Total (*N* = 31)	*p*
Gender (% of males)	56.5	50.0	54.8	1.000^b^
Mean age (years)	60.4 [19.45]	26.0 [7.21]	51.55 [22.90]	<0.001^c^
Mean time from the onset of symptoms (days)	7.6 [3.10]	4.5 [1.41]	6.81 [3.07]	<0.001^d^
Mean time from the last positive PCR (days)	1.8 [1.98]	3.5 [2.54]	3.03 [2.50]	0.145^c^
Fever on collection day (%)	69.6	25.0	58.1	0.043^b^
Respiratory symptoms^e^ (%)	95.7	67.5	87.1	0.043^b^
Gastrointestinal symptoms^f^ (%)	47.8	25.0	41.9	0.412^b^
Mean C‐reactive protein level^g^	79.1.4 [68.34]			
Mean ferritin level in plasma	457.2 [347.88]			
Abnormal leucocytes (%)	30.4			
Low lymphocytes (%)	73.9			
Low eosinophiles (%)	65.2			
High alkaline phosphatase (%)	14.3			
High alanine aminotransferase (%)	43.5			
Mean D‐dimer value^h^	2.2 [6.91]			
Mean fibrinogen value	4.7 [1.34]			
Low SpO2 level (%)	65.2			
High respiration rate (%)	78.3			

*Note*: Standard deviations are reported in brackets [].

^a^ Laboratory results of home‐treated patients were not available.

^b^ Fisher–Freeman–Halton exact test.

^c^ Independent‐samples Mann–Whitney *U*‐test.

^d^ Independent‐samples t‐test.

^e^ Cough, hoarseness, sore throat, or shortness of breath.

^f^ Diarrhea, vomiting, stomach pain, or nausea.

^g^ Results below the detection limit of 4.0 were set to 2.0 for calculations.

^h^ Results below the detection limit of 0.3 were set to 0.2 for calculations.

### 3.2 SARS‐CoV‐2 RNA in air

Overall, 258 air samples were obtained from 29 air collections (Table S3). The samples were divided into actively and passively collected samples based on the collection method (see methods and the results below for details). In total, 33 (12.7%) air samples from 12 (41.4%) collections were qRT‐PCR positive. The rate of positive home collections was 57.1% and of hospital collections 36.3% (*p* = 0.403, Table S2). Estimated copy numbers varied between 1.04 × 10^3^ copies/ml and 2.05 × 10^7^ copies/ml. All air samples were cultured, but no viable viruses were observed. The protocols and tests used to optimize the culturing protocol are described in the Supplementary Methods. Although five hospitalized patients (index patients P6, P7, P48, P56, and non‐index patient P5) used nasal canula oxygen or an oxygen mask during collection, which are considered aerosol‐generating procedures (AGPs), no positive samples were found during these collections. None of the patients used facemasks or respirators during their stay in the ward or in the ICU or during the measurements, which was according to the hospital policy as universal masking regarded only the staff and visitors.

#### 3.2.1 Active air sampling

Altogether, 154/258 (59.7%) air samples were collected with five different active air samplers, comprising (1) three simultaneous Andersen six‐stage cascade impactors (Andersen) with different sampling times, (2) a BioSpot 300p bioaerosol sampler (BioSpot), (3) a Button sampler (Button), (4) a Dekati PM10 cascade impactor (Dekati), or (5) a Dekati eFilter (eFilter). The time from the start of symptoms to air collection varied from 3 to 12 days. Positive samples were observed in 3/44 (6.8%) samples with the Dekati, 0/9 (0.0%) with the Biospot, 2/9 (22.2%) with the Button, 15/90 (16.7%) with the Andersen, and 0/2 (0.0%) with the eFilter (Table S4, results from home and hospital collections have been combined, as there was no statistically significant difference between the positivity rates for the collections). SARS‐CoV‐2 RNA was found in particles in the size ranges 0.65–4.7 μm and >7 μm in Andersen collectors, >10 μm and <2.5 μm in Dekati samplers, and <100 μm in Button samplers (Table 2; Table S3). On‐line particle concentrations measured with the eFilter on the COVID‐19 ward were in the range of 534–6608 cm^−3^ (3380 ± 2320 cm^−3^), and no clear particle emission events were observed.

**TABLE 2:** Characteristics of qRT‐PCR‐positive air samples with active air sampling

Place	Patient	Days from the onset of symptoms	Sampler	Sampling time (min)	Collected air volume (l)	Size fraction (μm)	Ct value N1 US CDC/(N Charité)
Hospital, cohort	P2	10	Dekati	195	3900	>10	36.64
	P40	4	Button	18	72	≤100	35.33
	P49	7	Andersen 1	10	283	1.1–2.1	36.45
				10	283	0.65–1.1	32.87
			Andersen 2	20	566	7.0–12.0	33.85/(37.47)
				20	566	3.3–4.7	36.31
				20	566	2.1–3.3	36.51
				20	566	1.1–2.1	33.77
				20	566	0.65–1.1	35.20
			Andersen 3	30	849	7.0–12.0	36.92
				30	849	3.3–4.7	33.12/(37.85)
				30	849	2.1–3.3	34.46
				30	849	0.65–1.1	35.20
	P54	7	Andersen 1	10	283	3.3–4.7	36.89
				10	283	1.1–2.1	33.76
			Andersen 2	20	566	2.1–3.3	36.61
			Andersen 3	30	849	3.3–4.7	36.70
Home	P10	2	Dekati	180	3600	1.0–2.5	34.84
				180	3600	<1.0	31.95
	P42	5	Button	21	84	≤100	35.09

#### 3.2.2 Passive air sampling

A total of 91 passive air samples (14 collections) were collected by deposition on open‐cell culture plates. The mean collection time was 0.69 h (range 0.3–3.0 h, SD 0.51), and the mean distance from the patient was 0.94 m (range 0.2–5.0 m, SD 0.84). Sampling points differed between home and hospital collections (*p* = 0.001, Table S2), but distances from the patient were similar (*p* = 0.398, Table S2). In total, 12 deposition sample (11.5%) in 8 (57.1%) collections were positive for SARS‐CoV‐2 RNA (Table 3). There was no statistically significant difference in the proportions of qRT‐PCR‐positive samples (*p* = 0.333) or collections (*p* = 1.000) between home and hospital (Table S2).

**TABLE 3:** qRT‐PCR‐positive passive air sampling (deposition) results based on the sampling place and distance from the patient. Mean Ct is reported based on N1 US CDC qRT‐PCR and copy numbers as copies/ml of original sample based on N Charité qRT‐PCR are reported in parenthesis when applicable

	Total			Hospital		Home	
	*N*	%	Mean Ct value (copy number)	*N*	%	*N*	%
Window sill	2/8	25.0	35.87	0/4	0.0	2/4	50.0
Table	5/42	11.9	33.31 (7.15 × 10^6^)	3/21	14.3	2/21	9.5
Behind the patient	1/7	14.3	34.53	0/3	0.0	1/4	25.0
Floor	3/29	10.3	36.09	2/26	7.7	1/3	33.3
In front of the face during talking, coughing, spitting, or breathing	1/13	7.7	NA^a^ (1.45 × 10^5^)	1/11	9.1	0/2	0.0
Shelf	0/1	0.0	NA^a^	NA	NA	0/1	0.0
Another room with closed door	0/4	0.0	NA^a^	0/3	0.0	0/1	0.0
<0.5 m	7/43	16.3	34.86 (5.40 × 10^6^)	3/28	10.7	4/15	26.7
0.5–1 m	2/20	10.0	35.84	2/14	14.3	0/6	0.0
1–2 m	1/15	6.7	34.37	0/7	0.0	1/8	12.5
>2 m	1/9	11.1	34.47	0/5	0.0	1/4	25.0

^a^ When mean Ct is reported as NA, N1 US CDC qRT‐PCR was negative. N Charité qRT‐PCR results are presented in the Supplement.

Thirteen experimental respiratory samples (coughing, breathing, talking for 2 min in front of an open‐cell culture plate, or spitting once onto an open‐cell culture plate) were collected from seven patients with a mean symptom day of 7.8 (range 5–8, SD 2.17). Out of all the respiratory samples, only one of the spit samples was qRT‐PCR positive (Table S3).

### 3.3 SARS‐CoV‐2 RNA on surfaces

We collected 252 surface samples, 182 (72.2%) of which were from the hospital and 70 (27.8%) from patients' homes. In total, 25/252 samples (9.9%) from 15/27 collections (57.7%) were qRT‐PCR positive (Table 4; Table S5). Viable virus was not detected in any of the 212 cultured surface samples. There was no difference in the proportion of positive samples between the four surface groups (*p* = 0.646, Table S2) or between home and hospital collections (*p* = 0.351, Table S2). For a given positive collection, there was no significant difference between finding the virus from the air or on surfaces (*p* = 0.344, Table S2).

**TABLE 4:** qRT‐PCR‐positive surface samples divided into four surface groups. Mean Ct is reported based on N1 US CDC qRT‐PCR

Surface	Total			Hospital		Home	
	*N*	%	Mean Ct	*N*	%	*N*	%
High‐touch surfaces							
Bed remote	1/2	50.0	37.22	1/2	50.0	NA	NA
Other high‐touch surfaces	4/11	36.4	30.07	2/5	40.0	2/6	33.3
Cell phone	3/26	11.5	36.7	1/18	5.6	2/8	25
Drinking glass	2/18	11.1	34.82	0/11	0.0	2/7	28.6
Computer	1/12	8.3	29.22	0/2	0.0	1/10	10.0
Door handle	0/28	0.0	NA	0/19	0.0	0/9	0.0
In total	11/97	11.3	33.31	4/57	7.0	7/40	17.5
Low‐touch surfaces							
Hospital equipment	2/9	22.2	32.87	2/9	22.2	NA	NA
Other low‐touch surfaces	1/6	16.7	36.26	1/4	25.0	0/2	0.0
Floor	4/22	18.2	35.63	4/16	25.0	0/6	0.0
Table	3/38	7.9	33.49	2/30	6.7	1/8	12.5
Bed rail	1/19	5.3	37.79	1/19	5.3	NA	NA
Air vent	0/2	0.0		0/1	0.0	0/1	0.0
In total	11/96	11.5	35.61	10/79	12.7	1/17	5.9
Toilet surfaces							
Toilet seat	1/14	7.1	NA^a^	1/11	9.1	0/3	0.0
Toilet flush button	2/18	11.1	37.92	1/12	8.3	1/6	16.7
Tap	0/11	0.0	NA	0/7	0.0	0/4	0.0
Toilet bowl	0/8	0.0	NA	0/8	0.0	NA	NA
In total	3/51	5.9	37.92	2/38	5.3	1/13	7.7
Other surfaces							
Staff/PPE	0/8	0.0	NA	0/8	0.0	NA	NA
Total	25/252	9.9	35.61	16/182	8.8	9/70	12.9

^a^ When mean Ct is reported as NA, N1 US CDC qRT‐PCR was negative. N Charité qRT‐PCR results are presented in the Supplement.

### 3.4 Effects of patient factors on environmental contamination

Positive air samples were found even when the index patient did not report any respiratory symptoms (2/3, 66.6%). However, there was a statistically significant connection between low oxygen saturation (SpO2) levels and SARS‐CoV‐2 RNA findings from surfaces, and a possible but nonsignificant connection between low SpO2 levels and RNA findings from the air (surface: *p* = 0.026, air: *p* = 0.098, Table S2). Toilet surfaces were qRT‐PCR positive in 33.3% (3/9) of cases when the index patient had GI symptoms and 0% of cases (0/9) when the index patient did not report any GI symptoms (*p* = 0.229, Table S2). No positive environmental samples were obtained if the saliva sample from the index patient was negative with both qRT‐PCRs. Positive surface samples were detected more often when there were multiple COVID‐19 patients in the ward/house during the sampling (*p* = 0.018, Figure S1). However, no statistically significant difference was detected for air collections (*p* = 0.845) (Figure S1). Possible but statistically nonsignificant associations were observed between positive environmental samples and an earlier symptom day, as well as an older age (Figure S1 and S1c). No statistically significant connections were found between air and surface qRT‐PCR results and laboratory results for index patients (Figure S2). No significant connections were detected between virus strain and environmental contamination (see Supplementary Data for details).

### 3.5 SARS‐CoV‐2 in saliva

Saliva samples were obtained from 26/31 index patients and 10 other patients on the ward. In total, 22/26 of the index patient samples and 8/10 of the samples from other patients were qRT‐PCR positive. RNA copy numbers varied between 1.65 × 10^3^ and 5.13 × 10^7^ copies/ml (mean 3.55 × 10^6^ copies/ml [SD 1.10 × 10^7^]). Six of the qRT‐PCR‐positive samples taken between symptom days 2 and 11 were also positive in virus culture (five of which were index patients). Culture‐positive samples had lower Ct values than culture‐negative samples (*p* < 0.001 (N1 US CDC), *p* = 0.019 (N Charité), Figure S3). Age showed a trend of positive correlation with copy number, but it was not statistically significant (Spearman's rho = 0.339, *p* = 0.106, Figure S5a). The mean copy number in the saliva of the index patients was 9.37 × 10^5^ copies/ml (SD 7.57 × 10^5^) in collections that had qRT‐PCR‐positive air samples and 7.74 × 10^6^ copies/ml (SD 7.26 × 10^6^) in collections where all air samples were qRT‐PCR negative (*p* = 0.536) (Figure S5e). The respective figures for surface collections were 5.61 × 10^6^ copies/ml (SD 1.52 × 10^7^) in positive and 1.54 × 10^5^ copies/ml (SD 1.85 × 10^5^) in negative collections (*p* = 0.291) (Figure S5e). No connections were observed between saliva culturing results and PCR from the environment: PCR‐positive air samples were detected in 40% (2/5) of the collections when the saliva of the index patient was culture positive on a collection day and in 44% (8/18) of the collections when saliva was culture negative. In surface collections, the same numbers were 60% (3/5) and 65% (11/17).

### 3.6 SARS‐CoV‐2 antibodies in serum samples

Serum samples were obtained from 21 hospital‐treated patients (13 index patients and six other patients on the ward) and four home‐treated patients (two index patients and two other patients). In total, 10 serum samples were positive for IgG or Nabs. Antibodies were detected at the earliest on symptom Day 3 (P13, positive with two IgG tests, Nab titer 80). Of the antibody‐positive patients, 9/10 were qRT‐PCR positive from saliva and one (P16, symptom Day 11, Nab titer 80) was also positive in viral culture. The index patients had Nabs against SARS‐CoV‐2 in five of the collections, and in four of these, PCR‐positive environmental samples were detected (active air samples in one (P49, Nab titer >640), deposition air samples in two (P41 and P43, Nab titers 40 and 10), and surface samples in two (P13 and P43, Nab titers 80 and 10)).

### 3.7 Transmission of COVID‐19 to family members

The spread of COVID‐19 within the family was examined by collecting saliva samples from family members of the five home‐treated patients and analyzing qRT‐PCR results and SARS‐CoV‐2 antibody levels. In two families that used protective measures, including respiratory protection (surgical mask or respirator) and intensified cleaning, no further infections were detected. One of these families used masks in common areas, but not in their own rooms behind closed doors. In another family, the bedroom was shared, and masks were used all the time. However, in three families that did not apply any protective measures or used only intensified cleaning, secondary infections were observed. In two families, all other family members were infected, and in one family, one out of three other family members was infected. (See Supplementary Material for details regarding measures used to prevent further infections).

## 4 DISCUSSION

This study detected considerable SARS‐CoV‐2 RNA contamination from both home and hospital environments. The virus was found in the air in particle size ranges of 0.65–4.7 μm, 7.0–12.0 μm, >10 μm, and < 100 μm in diameter (Table 2), supporting existing literature.^17^, ^19^, ^20^, ^22^ Our findings also support discoveries that normal respiratory activities generate infective particles even in the absence of AGPs,^2^, ^6^, ^64^, ^65^ and respiratory symptoms. Additionally, low oxygen saturation showed a connection with a higher possibility of SARS‐CoV‐2 surface findings and a potential connection with air findings, which could follow from increased particle generation due to respiratory stress. Most (83%, 15/18) of our positive air samples with known particle size were in particles smaller than 4.7 μm, which supports the findings that at least 85% of the viral load is emitted in aerosols smaller than 5 μm.^6^, ^20^, ^66^ This is in line with the fact that particle generation produces a distribution which form depends on the activity that is causing the particles. In human respiratory activities, generated particles are mainly small, under 5 μm in dry size distribution.^67^, ^68^

A previous study showed that a high sampling flow rate increases the success rate in detecting SARS‐CoV‐2 from air samples.^21^ When 50 L/min air samplers were used, no positive samples were detected, but when sampling flowrate was raised to 150 L/min 72% of samples were positive.^21^ In our study, SARS‐CoV‐2 was detected from the air with a minimum collection period of 10 min (Andersen's impactor) and a minimum air volume of 72 L (Button sampler). With an average respiratory rate of 14/min and volume of 0.5 L/breath, this would mean exposure times of 40 min (Andersen) and 10 min (Button) for the examined virus variants (alpha and undetermined VoC (Andersen), as well as non‐VoC (Button)). Current safety guidelines use 15 min exposure time regarding contact tracing.^69^ Our results, although limited due to the low number of collections and only qRT‐PCR findings, support concerns that a shorter exposure time should be considered, at least for close contacts.^70^ Overall, the exposure risk is cumulating with time and no limit to zero risk can be determined. The risk for infection depends on the concentration exposed to (depending on ventilation and produced quanta) as well as persons immunity.^27^, ^71^, ^72^, ^73^ Virus variants such as the delta and omicron variants seem to lower the exposure time needed for infection, following estimated higher viral load in the presence of the delta variant^74^ and increased transmissibility of omicron variants possibly due immune evasion.^75^, ^76^ Also, already low infectious doses have been shown to cause infection in an animal model.^38^, ^77^ A previous study did not observe significant differences in environmental contamination prevalence or Ct values between alpha and omicron B.1.1.529, which could further point toward a higher receptor binding affinity and immune escape properties of omicron variant^78^ and enable the use of studies carried out with previous variants also when evaluating the environmental burden of newer variants.

Our results from respiratory activities demonstrated that 0.5–2 min of activity did not produce enough virus to be detected with qRT‐PCR with this methodology, even from a close distance of 10 cm. However, it should be noted that the open deposition collection method for both respiratory activities and passive air (deposition) samples is highly dependent on the success of the impactation and the flow field near the collection surface,^79^ being susceptible for example to head movements and indoor air flows. Still, a quite high proportion (11.5%) of passive air samples was positive for SARS‐CoV‐2 RNA. This supports the role of aerosol particle deposition as a source to surface contamination as previous studies have suggested.^17^, ^80^ The deposition offers more gentle collection method and possibility to collect directly to the cells or cell media possibly allowing more viral preservation compared to active sampling. This could at least partly explain why viral findings were seen in our work and in previous work^17^ when active aerosol samples remained negative.

Multiple positive air samples were collected from a large (655.25 m^3^) mechanically ventilated hospital hall (Figure 1), even when there were only two patients. Overall, larger spaces are considered safer than small ones due to the larger air volume per person.^81^ However, it seems that also larger indoor spaces may form a risk environment if occupied by an infected person for a prolonged time period.^82^, ^83^, ^84^ In our study, all patients in the ward were COVID patients. However, in many countries, COVID‐positive patients have been separated from COVID‐negative ones with just curtains and distance. As hospital‐acquired infections have been a significant part of overall infections and deaths,^85^, ^86^, ^87^ it is important to reduce the risk of infections in hospital wards. It should be noted that the infective aerosol particles may still generate an infection risk even when larger space allows more dilution with increasing distance, as shown by Karan et al.^88^ Our findings were mainly from a close distance similar to a previous study that saw higher probability to environmental findings inside 2‐meter range,^24^ even though the risk for infection especially in prolonged exposure remains also further away.^27^, ^28^

It is interesting that the proportion of the positive samples was similar in hospital and home even when the ventilation was more efficient in the hospital and patients have later symptom day. This may be due to more patients in the same room or higher overall viral load which has been associated with more severe disease and higher age in previous studies.^89^, ^90^ We observed a trend for an older age being associated with a higher viral load (Figure S5a) and a larger number of positive surface samples but confirming this would require further studies with a larger sample sizes. In earlier studies, higher viral loads have been associated with an increased probability of viral transmission.^91^, ^92^ Possible reasons for the relationship between age and infectivity include reduced saliva production, differences in mucus viscosity and salivary immunoglobulins,^93^ increased expression of the ACE2 receptors needed for cell entry of SARS‐CoV‐2,^94^ thinning of the epithelium,^95^ and impairment of the immune response with age.^96^

Toilet surface samples were positive only when the index patient‐reported GI symptoms. Infectious SARS‐CoV‐2 has been recovered from urine and stool samples,^97^ and flushing of the toilet and vomiting can generate aerosols, which will later deposit on the surfaces.^98^, ^99^ This risk should be noted in both environments and toilets should not be shared with non‐COVID patients, if possible. Other more frequently qRT‐PCR‐positive surfaces included highly touched personal items, hospital equipment, and the floor, which is in line with the previous findings.^18^, ^19^, ^34^ Even though RNA may persist on surfaces for some time, RNA findings most likely result from contamination on the same day due to daily cleaning.

The building body of evidence supports airborne route predominance for SARS‐CoV‐2 transmission,^1^, ^2^, ^3^, ^4^, ^14^ and an animal study indicates that aerosol inoculation is a more efficient route and causes more severe pathology and higher viral loads.^100^ Fomites have not been proven to serve as the sole or primary vehicle of transmission.^101^ The probability for surface transmission is estimated to be likely rare, generally less than 1 in 10 000, and the disease manifestation milder.^102^ The environmental samples that commonly presented infectious virus in previous studies were mainly in direct contact with infected patients' mucus membranes, or saliva or sputum secretions (e.g., nasal prongs, nasal canula, used tissue, patients mask, and endotracheal tube).^25^, ^38^ In this study, families that took protective measures (including isolation of the infected family member) and respiratory protection (surgical masks or FFP2 respirators) were able to prevent further infections even when qRT‐PCR‐positive samples were collected from both surfaces and air. However, in a household where all surfaces were cleaned many times a day but no respiratory protection was used, all family members became infected. This supports the importance of air hygiene, including also portable air cleaners as a supportive method as shown in previous studies,^15^, ^28^ and also encourages control of infection spread in homes. Similar findings supporting the use of masks, isolation with closed door, and opening windows in home environment were found to lower the risk of contamination in the work of Picard et al.^103^ Overall our results indicate that transmission may happen through several transmission routes as supported also in previous systematic review.^20^ Infection control is even more important with VoC strains that feature a higher rate of household transmission.^104^

To better understand the infectivity and state of the infection compared to the environmental findings, we collected saliva and serum samples. SARS‐CoV‐2 was cultured from saliva during symptom Days 2–11.^42^ SARS‐CoV‐2 RNA was detected in the saliva of patients who had already formed IgG and NAbs, which align with previous findings of prolonged RT‐PCR‐positivity.^105^, ^106^, ^107^ In addition, the saliva of P16 on symptom Day 11 was still positive in virus culture, even though the patient had NAbs. Moreover, we obtained positive air and surface samples when the index patient had a positive IgG result and NAbs, which agrees with the findings of Lei et al.^108^ This contradicts the suggestion that NAbs solely could be a reliable marker for non‐infectivity^78^ ^.^ In the view of infection control, we agree with Lei et al.,^108^ Tan et al.^109^ and Wölfel et al.^110^ that the risk for exposure can remain after the patient starts to seroconvert and possibly improve clinically. As seroconversion seems an unreliable marker for viral clearance, other means, such as an antigen test, should be used to assess the infection risk before ending precaution protocols.^111^, ^112^

Showing viable virus from environmental is highly demanding. Previous studies have presented a few possibly positive findings.^10^, ^11^, ^12^, ^13^, ^36^, ^37^, ^38^, ^66^ However, the findings of those studies have been under criticism as none of them has been able to show clear CPE with significant decrease in Ct values leaving questions if the CPE was truly induced by SARS‐CoV‐2. Despite the culturing attempts of all 258 air samples and 212 surface samples, we were unable to detect viable virus from the environment. Also, only 20% of qRT‐PCR‐positive saliva samples of symptomatic patients were positive in cell culture. We did several attempts to optimize the culture protocol (Supplementary Methods) and performed qRT‐PCR on culture media whenever the slightest sign of CPE was detected. Explanations for our negative results are that there was no infectious virus in the environment at the time of the samplings or that current methods were not sensitive enough to detect it. As SARS‐CoV‐2 is efficiently transmitted in hospital wards and between family members,^113^, ^114^ even from quarantine room to another via corridor and similarly timed door opening,^115^ we conclude that the methodology is likely too unsensitive.^116^ A significant loss of infective viruses in air sampling has also been demonstrated,^117^, ^118^, ^119^, ^120^, ^121^, ^122^, ^123^ and current impaction and impingement sampling methods have low collection efficiency for small, nanometer‐sized particles.^121^, ^122^ Sousa et al. managed to show SARS‐CoV‐2 related PFU findings with electrostatic air sampler, which may provide a more gentle collection method, however, even they observed rapid and significant inactivation of the virus during collection.^12^ The absolute collection efficiencies of the samplers are unknown and should be addressed in future research.^123^ Even though detection of viral RNA in the air and on the surface does not necessarily mean an infectious virus, negative culturing results do not rule out the infection risk either. Due to these difficulties and the high transmission rates, we consider already RNA findings to be interpreted as a possible infection risk. In the future, sampling methods and devices should be developed to better preserve the viability and infectivity, for example mimicking the humidity and airflow of the airways to avoid mechanical stress and utilizing direct collection onto the cells or culture medium to avoid losses during transport. In an ideal situation, the future sampling methods would also allow on‐site recognition of the viruses from air as well as from surfaces.^124^ Also, additional methods (e.g., virus sequencing) that can be applied to a large number of samples should be utilized and further developed in future studies on environmental transmission.

Overall, this study combined a large number of environmental samples and detailed patient data to more comprehensively understand environmental contamination and the effect of patient‐dependent factors. The patient material was representative regarding symptoms and laboratory results for COVID‐19.^125^ In addition to the hospital environment, we collected samples from homes where symptoms are generally less severe, the time from the onset of symptoms is shorter, and air conditioning is different from that of a hospital. Recent findings suggest that the environmental contamination is rather similar between the first variants and omicron,^78^ suggesting that the results from the earlier variants can still be considered to provide valid information for the current situation.

Our study also has some limitations. Even though the overall number of our samples is quite high compared to previous studies, it is still limited in the statistical aspect and only able to detect major differences and associations. As environmental sampling is time consuming and resource intensive, making it challenging to achieve a statistically large enough sample size, it is important to combine findings from several different studies for more detailed analysis. We only conducted environmental sampling at a single time point. In the future, a longitudinal examination could enable a more accurate examination of the effects of the course of disease for environmental contamination. The mean time from the onset of symptoms until sampling varied between homes and hospital and may affect the results. However, as the infection requiring hospital treatment is more severe, the viral loads may stay high longer,^112^, ^126^ and accordingly, no major differences were detected in viral load in saliva in our study between homes and hospital patients. This would provide rather similar expectation for environmental contamination as Buonanno et al examined.^14^ Also, patients generally arrive to the hospital at the later stage of the disease (excluding hospital‐acquired infections that were not detected in this dataset) which makes our dataset suitable to represent the real situation between homes and hospitals. In addition, we only measured the IgG and NAb response, but viral secretion from mucus membranes can continue if the IgA response is weak.^127^ The IgA immune response should thus be examined further in upcoming studies. The qRT‐PCR results might include some uncertainty due to the differences in the texture and fluidity of saliva and should be considered as estimates. As many samples were collected from a large patient hall, it is possible that some observed viruses might have originated from other than the index patient. However, most of the surface samples were from patient‐specific surfaces, and aerosols are known to concentrate near the source,^71^ indicating that most of the positive samples are expected to be produced by the index patient. Particle size cutoffs in Andersen samplers might be slightly higher than estimated, as the amount of liquid used in the sampling was slightly smaller than recommended due to practical reasons. Finally, we strongly suggest developing a new, more sensitive methodology for assessing the virus viability to better assess transmission mechanisms.

## 5 CONCLUSIONS

This study found SARS‐CoV‐2 RNA from air samples in wide range of different‐sized particles during normal respiratory activity from both home and hospital environment. We observed positive air samples from a large ventilated patient hall in collections corresponding to 10 min of normal respiration, although current restricted air sampling techniques may have caused some virus loss. We also detected SARS‐CoV‐2 RNA‐positive air and surface samples after patients had developed antibodies. These results highlight the need for appropriate infection control against airborne and surface transmission routes in both environments, even after antibody production has begun.

## AUTHOR CONTRIBUTIONS

Lotta‐Maria A. H. Oksanen, Jenni Virtanen, Antti‐Pekka Hyvärinen, Vinaya Venkat, Kirsi Aaltonen, Ilkka Kivistö, Lev Levanov, T.S., S.S., L.M, N.S.A., N.R., E.S, and S.L., designed the study and developed the methodology. Lotta‐Maria A. H. Oksanen, Jenni Virtanen, Vinaya Venkat, Kirsi Aaltonen, Svetlana Sofieva, Nina S. Atanasova, Aurora Díaz Pérez, Noora Rantanen, Joel Kuula, and Julija Svirskaite collected the samples. Jenni Virtanen, Vinaya Venkat, Kirsi Aaltonen, Ilkka Kivistö, Aurora Díaz Pérez, Leena Maunula, and Joel Kuula, analyzed the samples. Lotta‐Maria A. H. Oksanen and Jenni Virtanen analyzed and visualized the data. Lotta‐Maria A. H. Oksanen and Jenni Virtanen wrote the original draft. All authors revised and edited the manuscript. Tarja Sironen, Nina S. Atanasova, Leena Maunula, and A.G. supervised the study. Lotta‐Maria A. H. Oksanen, Antti‐Pekka Hyvärinen, Tarja Sironen, Nina S. Atanasova, Enni Sanmark, Veli‐Jukka Anttila, Lasse Lehtonen, Maija Lappalainen, and Ahmed Geneid were responsible for project administration, funding, and recourses.

## CONFLICT OF INTEREST

The authors declare no competing interests.

## Supporting information


Appendix S1

Appendix S2

## Data Availability

All data are included in the article or its online Supplementary Material and are available from the corresponding author upon request.
