# Supplementary material for: SARS‐CoV‐2 indoor environment contamination with epidemiological and experimental investigations
Source: Indoor Air. 2022 Oct 24;32(10):e13118. doi: 10.1111/ina.13118 (PMC9828560; doi:10.1111/ina.13118)
Supplement: Supplementary file 1 — Appendix S1 [file INA-32-0-s001.docx]

# Supplementary Materials for manuscript SARS-CoV-2 indoor environment contamination with epidemiological and experimental investigations

**All supplementary tables are in a separate Excel-file and can be accessed through**

[**https://figshare.com/articles/dataset/Supplementary_tables/20560722**](https://figshare.com/articles/dataset/Supplementary_tables/20560722)


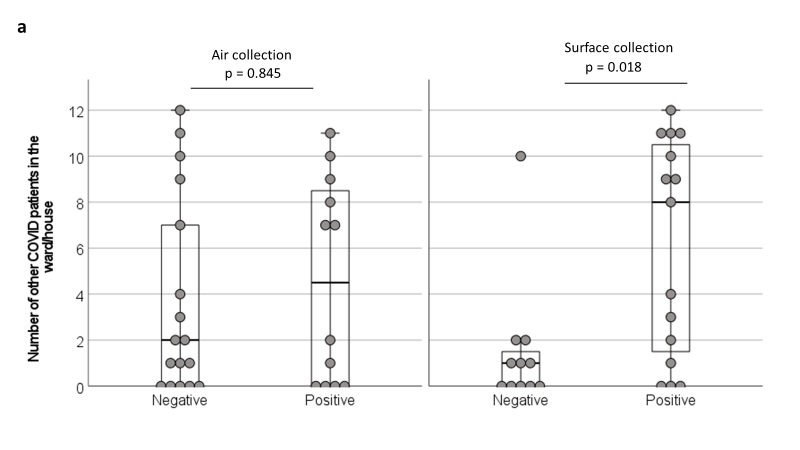

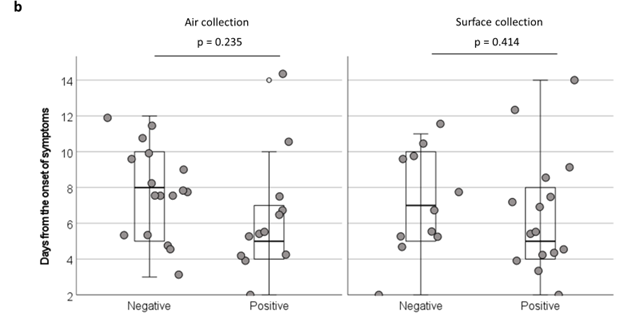


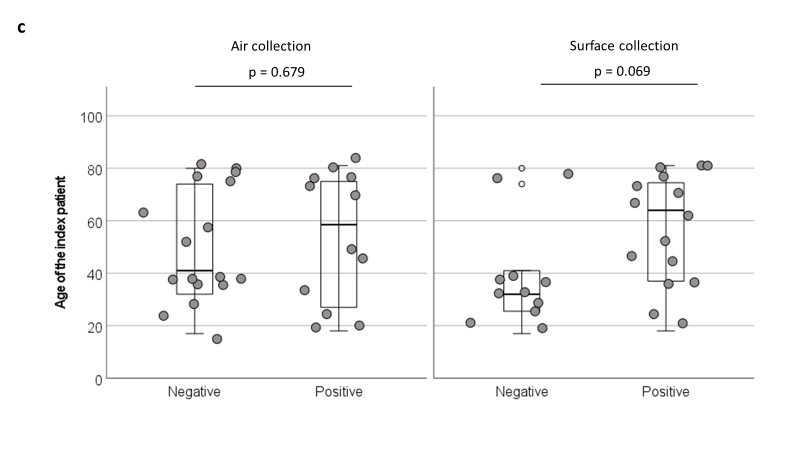


**Fig. S1 Connections between environmental contamination and patient characteristics.** **a** Number of COVID patients in the ward/house. **b** Days from the onset of symptoms. **c** Age of the index patient. Figure was created with SPSS IBM Statistics version 27 ^1^.


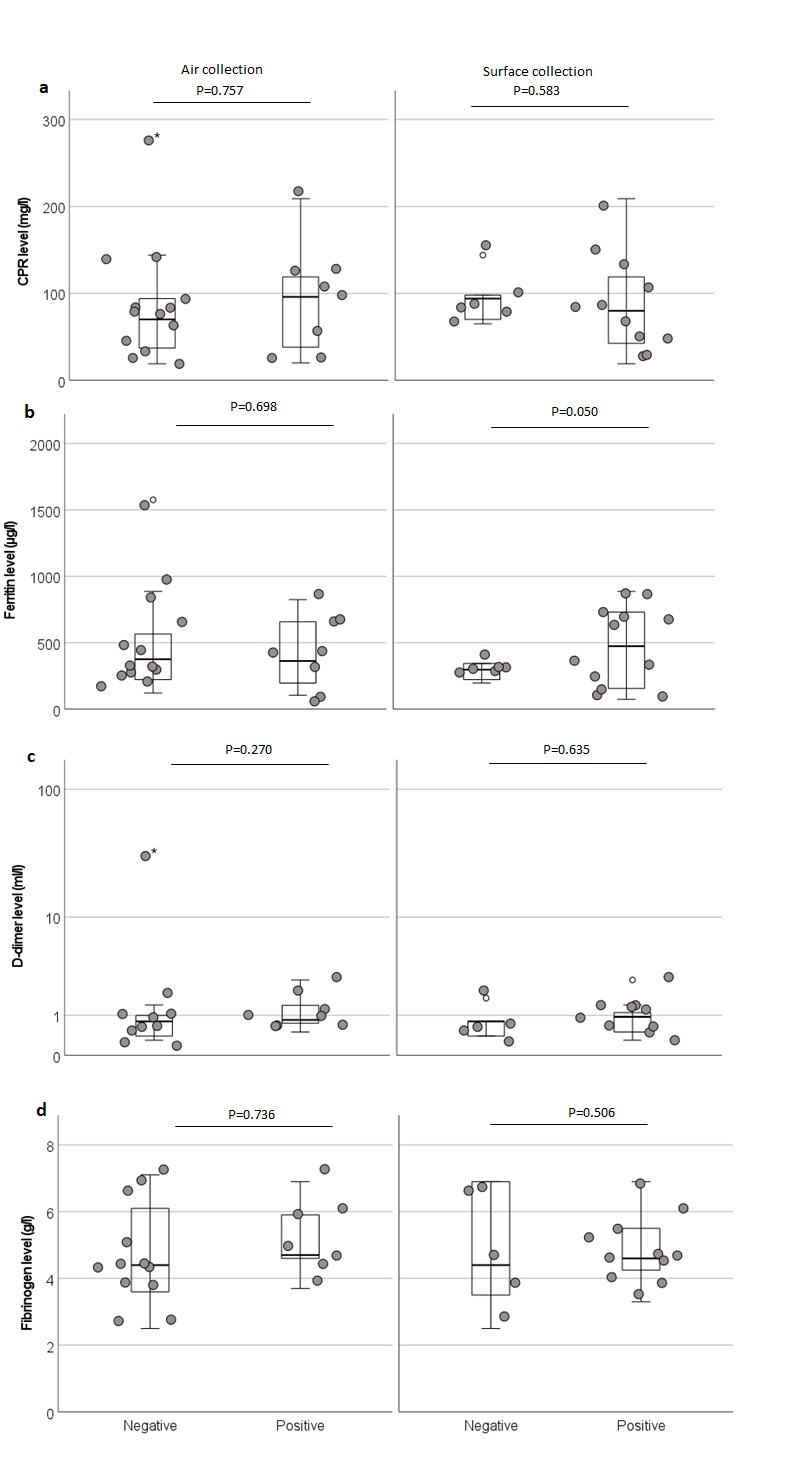


**Fig. S2 Environmental contamination compared to index patient’s laboratory results**: **a** CRP, **b** ferritin level, **c** D-dimer level, and **d** fibrinogen. Figure was created with SPSS IBM Statistics version 27^1^.

###
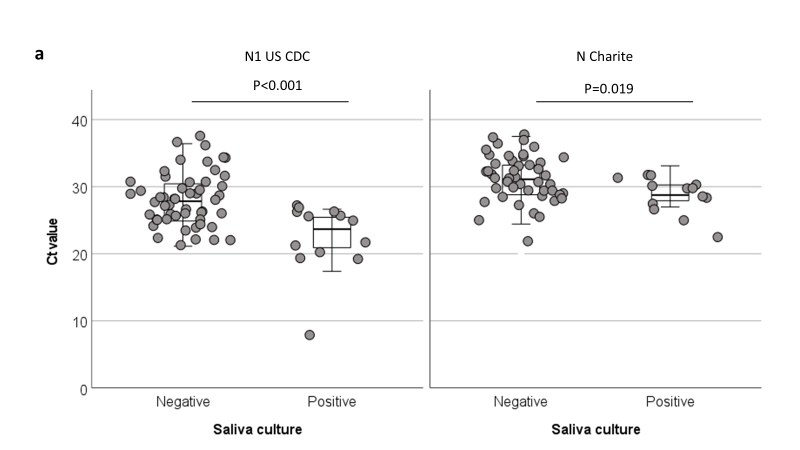


###
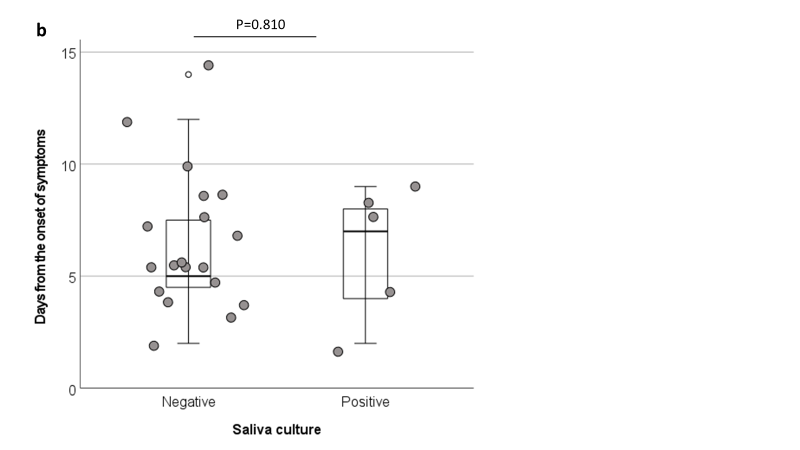


**Fig. S3 Saliva culture results. a** Comparison between saliva culture and PCR results. **b** Comparison between saliva culture results and days from the onset of symptoms. Figure was created with SPSS IBM Statistics version 27^1^.


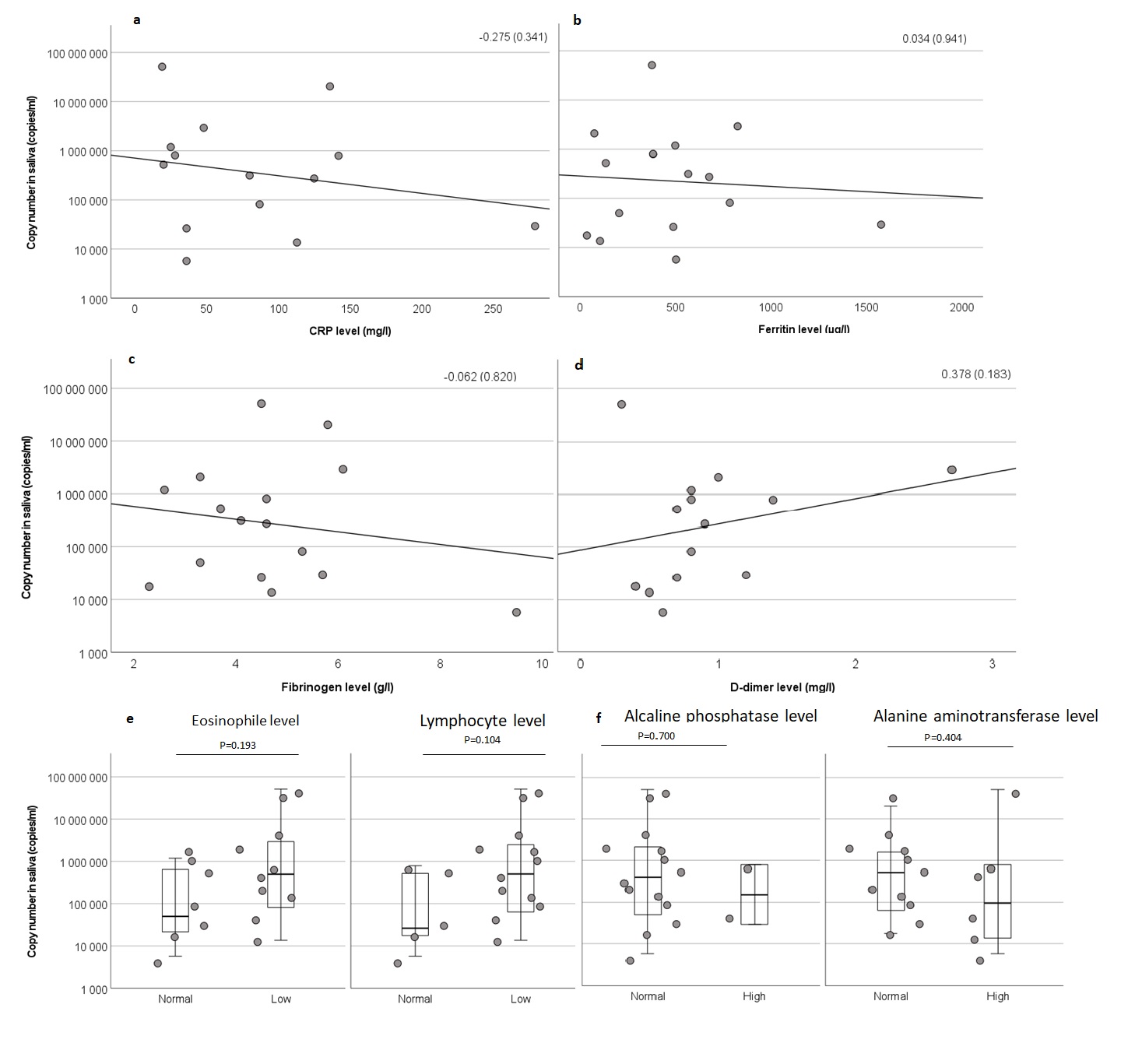


**Fig. S4 Correlation between SARS-CoV-2 RNA copy number in saliva and laboratory results: a** C-reactive protein (CRP), **b** ferritin, **c** fibrinogen, **d** D-dimer, **e** lymphocyte and eosinophile levels, and **f** alkaline phosphatase and alanine amino transferase levels. Spearmans’ rho values (with corresponding p-values) are indicated in (**a-d**) and p-values of Independent-Samples Mann-Whitney U-test in (**e-f**). Figure was created with SPSS IBM Statistics version 27^1^.


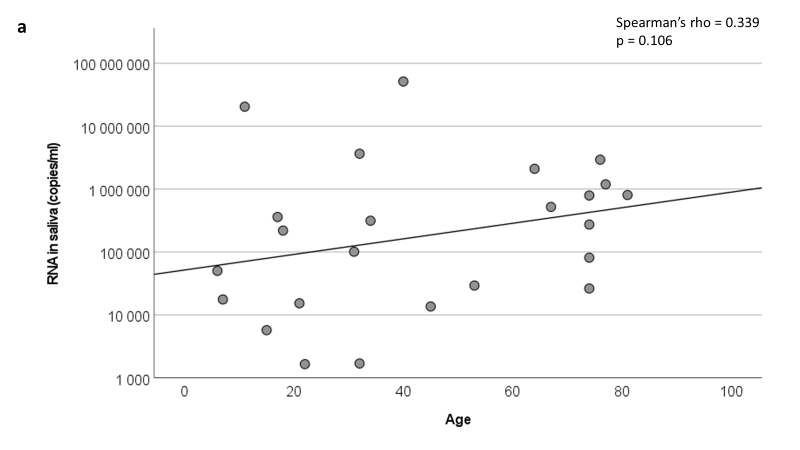

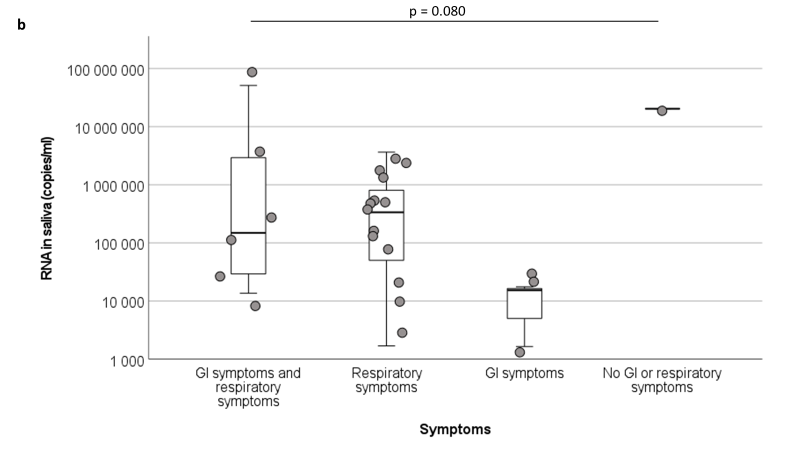

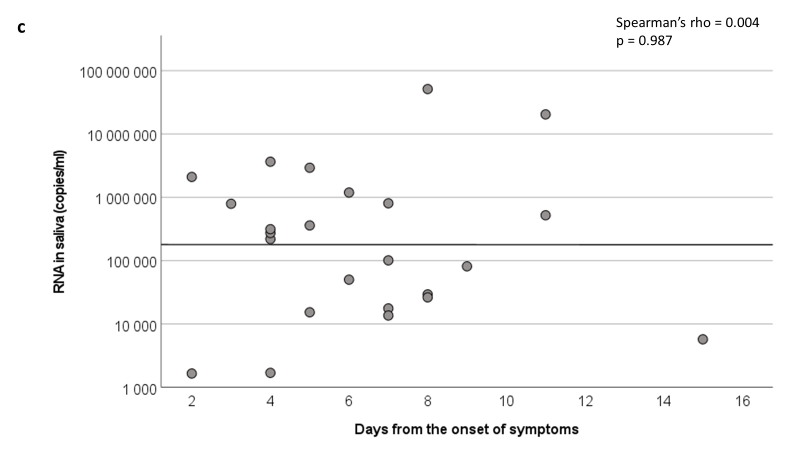

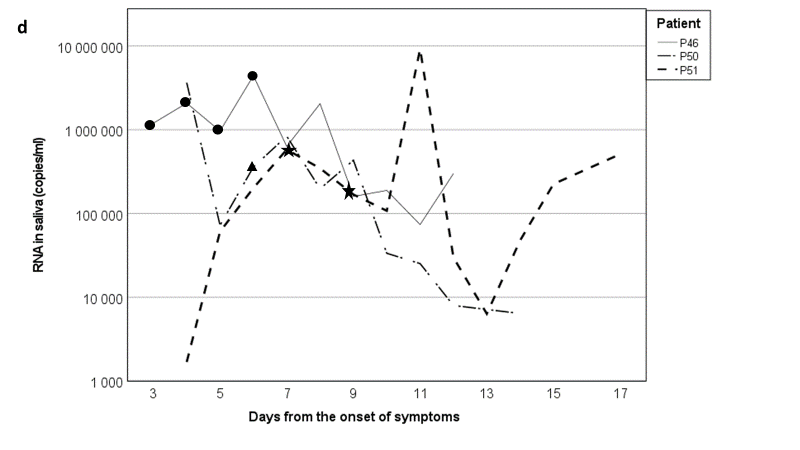

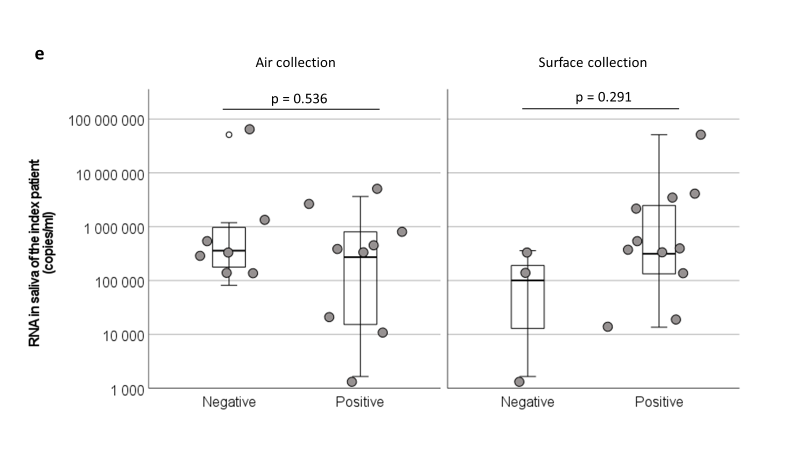


**Fig. S5 Comparisons of SARS-CoV-2 RNA copy number in saliva with other results and patient characteristics. a** Correlation between copy number and age. **b** Copy number grouped by symptoms (GI = gastrointestinal). **c** Correlation between copy number and time from the onset of symptoms. **d** Changes in the copy numbers of three home-treated patients who provided follow-up samples. Days, when the virus culture was also positive are marked with dots (P46), triangles (P50), and stars (P51). **e** Copy number in the saliva of the index patient grouped by qRT-PCR results from air and surface collection. Figure was created with SPSS IBM Statistics version 27^1^.

### Patients P46, P50, and P51 provided daily follow-up saliva samples until symptom days 12, 14, and 17, all of which (33/33) were qRT-PCR positive and seven of which were culture positive (Table S6). No statistically significant connections were found between copy number and the patient’s laboratory results (Fig. S4), gender (p = 0.312), symptoms (Table S2, Fig. S5b), or time from the onset of symptoms (Spearman’s rho = 0.004, p = 0.987, Fig. S5c). The copy number in saliva displayed an unsteady decline in two of the three patients who provided follow-up samples (Fig S5d).


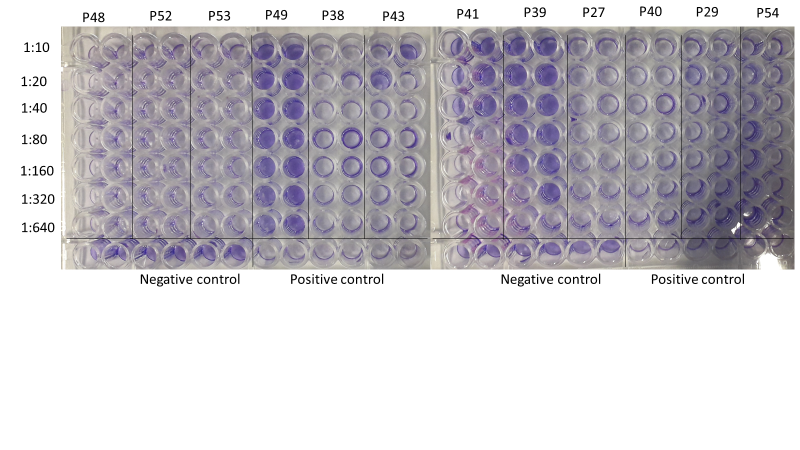


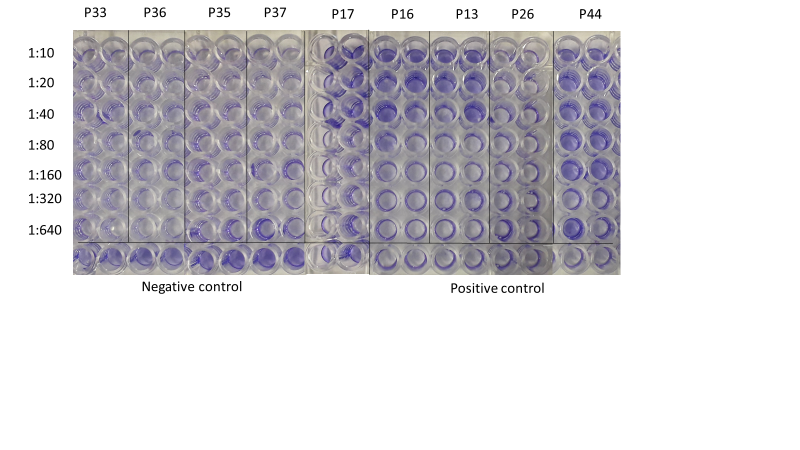


**Fig. S6 Microneutralization results against Fin/20 strain with serum dilutions from 1:10 to 1:640.** Positive control is the virus without serum and negative control is the cells without serum or the virus. Violet color indicates living cells which means that virus has been neutralized.

### Environmental contamination and virus strain

### The virus variant was determined in seven index patients during 2021, five of whom were infected with alpha variant, one with an undetermined variant of concern (VoC), and one with a non-VoC strain. The remaining cases were considered as non-VoC, as no VoC strains had yet been detected in Finland at the time of the collection. The mean RNA copy number in saliva was 1.12 x 107 (range 1.69 x 103 to 5.13x 107, SD 2.25 x 107) in patients with a VoC strain and 1.71 x 106 (range 1.65 x 103 to 2.04 x 107, SD 4.89 x 106) in patients with a non-VoC strain (p = 0.649). Altogether, 80% (4/5) of collections had positive surface samples when the index patient had a VoC strain and 52.6% (10/19) when the index patient had a non-VoC strain (p = 0.358). The respective results for the air collections were 50% (3/6) with a VoC strain and 42.9% (9/21) with a non-VoC strain (p = 1.000).

### Safety measures used in families of home-treated patients

### 1) In family of P11, no specific measures were taken to isolate the patient from the rest of the family and no mask or respirators were used. Index patient had very mild symptoms and was PCR negative from saliva on the collection day. Saliva sample from a highly exposed family member was PCR positive, but the family member didn’t have any symptoms.

2) In family of P33, patient was constantly separated from other family members right after the onset of symptoms. He stayed in separated room with closed door and used separated bathroom. Family members used FFP2-respirators and protective gloves. Index patient had fever and respiratory symptoms, and the culture and PCR-results from saliva were positive. Environmental contamination was detected both in surfaces and air of the patient’s room. Saliva samples as well as SARS-CoV-2 antibody tests from all family members were negative.

3) In family of P37, no specific measures were taken to isolate the patient from family. Additionally, no respiratory protection was used. Index patient had mild respiratory symptoms, but no fever. Index patients’ saliva was PCR positive but culture negative and no PCR positive environmental samples were detected. Saliva sample of one of the two tested family members was positive with both PCRs and family member had mild respiratory symptoms.

4) In family of P46, family member and patient used protective measures including all-time use of surgical mask and intensified cleaning. Saliva of the index patient was positive in PCR and virus culture but all the samples from the environment stayed negative. Saliva samples from the family member were negative. Despite of the high positivity in patients’ saliva, no notable transmission into environment occurred. Family member didn’t develop COVID-19 symptoms at any point after the collection, antibody samples were not obtained.

5) In family of P50 and P51, intensified cleaning was used, but no respiratory protection was obtained to protect family members from the infection. First infected member (a child) infected all other family members. A child had a high fever and respiratory symptoms. Other family members got their first symptoms three to four days after first symptoms of the child. Fecal samples were collected from two cats who also developed respiratory symptoms at the same time with other family members when their symptoms were already settled, but these samples were PCR negative. PCR-positive air and environmental samples as well as culture positive saliva samples were collected from both adults.

### Supplementary methodology: Protocol optimization

SARS-CoV-2 ability to infect VE6 cells in room temperature

To optimize passive air sampling protocol, the ability of SARS-CoV-2 to infect Vero E6 (VE6) cells in room temperature (RT) outside incubator and major differences between collecting samples to living cells versus collecting them to growth media which was transported to cells later in the laboratory were tested VE6 passaged SARS-CoV-2/Finland/1/2020 virus strain (Fin/20, passage 8, titer 10^6^ PFU/ml based on end-point titration) ^4^. 10-fold dilution series of the virus (10 000-1 copies of infectious virus) were pipetted into three 6-well plates of VE6 cells and into 35/10 MM cell culture dishes without cells containing 1 ml culture media (MEM). One of the 6-well plates was put straight to the incubator (37 °C), one was incubated in RT for 2 h (corresponding the time the cells have to be in RT during hospital collection) and one was kept in RT for 2 h and media was changed before putting into 37 °C. Cell culture dishes were kept at RT for 1 h, virus containing media was poured into the 50 ml falcon tube, which were put into the container with cold accumulators for 1h to imitate the transportation from the hospital and cultured in 6-well plates. Cells were grown for 4 days and checked for cytopathic effect (CPE).

CPE was observed in wells with approximately 1 copy of infectious virus in all cases indicating that cells were also infected at RT. When the virus was added to Petri dishes with MEM and transferred to cells later, CPE was also detected with approximately 1 copy of infectious virus, indicating that SARS-CoV-2 stayed stable in MEM for the time needed for transport into the lab. This is also supported by earlier studies reporting a good stability of SARS-CoV-2 in transport media^5^. One should note, however, that this test doesn’t effectively describe the situation with non-cell culture adapted strains and hence can’t be used to estimate the sensitivity of the culturing protocol and was only used to compare the protocols to each other.

Stability of SARS-CoV-2 on MCE and gelatin filters

SARS-CoV-2 stability on two filters used for Button sampling was tested by pipetting a dilution series of 10 000, 1000, 100, 10, 1, and 0 infectious virus particles in 10 µl onto MCE filters and gelatin filters (Sartorius Stedim Biotech). MCE filters were incubated at RT for time points of 15 min, 30 min, 1 h, 1.5 h, and 2 h and gelatin filters for 30 min after which the filters were moved to tubes with 3 ml of culture media. Tubes were kept in a container with cold accumulators for 1 h as before to imitate the sample transportation into the laboratory. MCE filter tubes were vortexed for 1 min at low speed. Medias from all the filter samples were poured to 6 well plates of VE6 cells and grown at 37 °C for 4 days.

Results are presented in Table S8. 30 min after adding the virus onto the filter, infectious virus was cultured from gelatin filter with approximately 1 copy whereas with MCE filter, the smallest virus amount was 1000 copies. After 1h, no infectious virus could be cultured from MCE filter indicating that virus inactivates on the filter relatively fast. Based on these results, gelatin filter was selected for collections over MCE filter and the collection time of approximately 30 min was used to maximize the changes of collecting enough virus from the air and culturing infectious virus before it is inactivated on the filter. One should note that in this set up, the virus was added onto the filter all at once, whereas in the hospital it can be assumed to be collected gradually. This set up also doesn’t compare the ability of the filters to collect the virus or tell if the stability is different in the collector with a heavier airflow.

Selecting PCR protocol

N Charité PCR was initially used due to being routinely used in the laboratory. After reports about more sensitive options being available, US CDC N1 (US CDC)^3^, China CDC N (China CDC)^6^, Institut Pasteur RdRP IP4 (IP4), and Institut Pasteur RdRP IP2 (IP2)^7^ were compared to N Charité PCR^2^. RNA extracted from strains C1P1^8^, VoC1^9^, and SARS-CoV-2/Finland/1/2020^4^ as 1:10, 1:100, 1:1000, and 1:10 000 dilutions was used in protocol comparison. All the PCRs were performed with TaqMan Fast Virus 1-Step Master Mix (ThermoFisher) according to fast cycling mode and annealing temperatures of 55 °C (US CDC), 58 °C (N Charité, IP2, and IP4), and 63 °C (China CDC). IP2 and IP4 were done as multiplex. All PCRs were performed with Stratagene Mx3005P (Agilent Technologies).

Ct-values are presented in Table S9 and Fig. S5. On average, Ct-values compared to N Charité were 5.0 cycles lower (SD 0.84) with US CDC, 1.5 cycles higher (SD 1.04) with China CDC, 2.4 cycles lower (SD 2.98) with IP4, and 1.9 cycles lower (SD 1.36) with IP2. Samples had gone through one extra freeze-and-thaw-cycle for China CDC, IP4, and IP2 PCRs which might slightly affect the Ct-values. US CDC PCR, which gave the lowest Ct-values, was also tested for specificity with MERS RNA and four SARS-CoV-2 negative saliva samples which all were negative. Due to these results and results by Etievant et al.^10^ US CDC PCR was considered to be most sensitive and specific enough to be used in the project in addition to N Charité PCR.


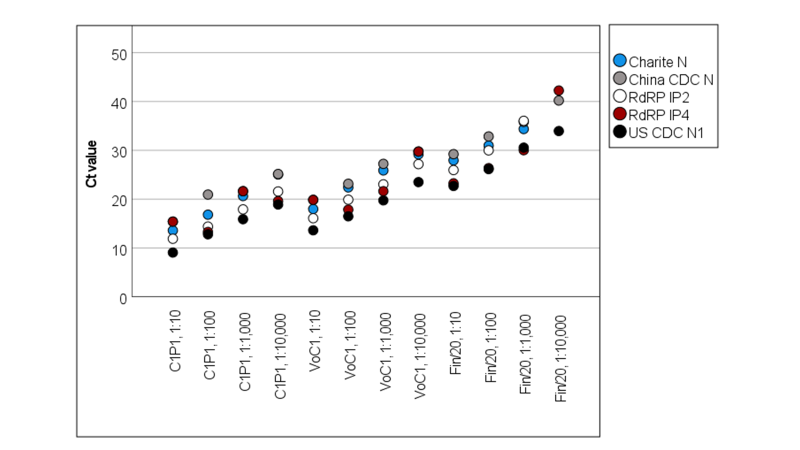


**Fig. S7 Ct-value comparison of five PCR protocols:** N Charité, US CDC N1, China CDC N, Institut Pasteur RdRP IP4, and Institut Pasteur RdRP IP2. Figure was created with SPSS IBM Statistics version 27^1^.

Optimizing saliva collection and handling

The inhibitory effect of saliva in PCR and virus culture was estimated with three saliva samples from a SARS-CoV-2-negative person. Saliva 1 was taken in the morning before eating or drinking anything, saliva 2 right after eating, and saliva 3 after eating and rinsing the mouth. Dilution series (10^-1^-10^-4^) of the Fin/20 strain was prepared in all saliva samples and PBS as a control. All the dilution series and unspiked samples were cultured in VE6 cells in 6-well plates by adding 100 µl of the sample to 900 µl of media, incubating 1 h, washing the cells with culture media, and adding 3 ml of fresh media. Dilution series in saliva 1 was also cultured in two additional ways. First, samples were centrifuged at 16 100 g for 5 min and the supernatant was cultured as above. Second, 75 µl of spiked saliva was added to 3 ml of media and the sample was left to the cells. Cells were grown at 37 C for five days and checked for CPE and estimated for contamination caused by other microbe flora in the mouth. RNA was extracted from all spiked saliva samples and PBS controls with Trizol (100 µl of sample to 900 µl of reagent) and tested with N Charité PCR.

No difference was detected in culturing sensitivity between virus diluted in different saliva samples (Table S10) indicating that food in the saliva didn’t notably decrease sensitivity in virus culture. Centrifuging the saliva before culturing got rid of fungal contamination but also appeared to slightly decrease sensitivity. Hence, centrifugation was not used in the study as other culturing protocol appeared more sensitive despite the fungal growth. Virus diluted in saliva 2 taken after eating had higher Ct-values in PCR than other saliva samples or virus diluted in PBS which indicates that food remains can inhibit PCR (Fig. S6). No notable difference was detected between virus diluted in saliva taken before eating and after rinsing mouth as compared to virus in PBS. Because of these results, patients were asked to rinse their mouth before sampling whenever possible.


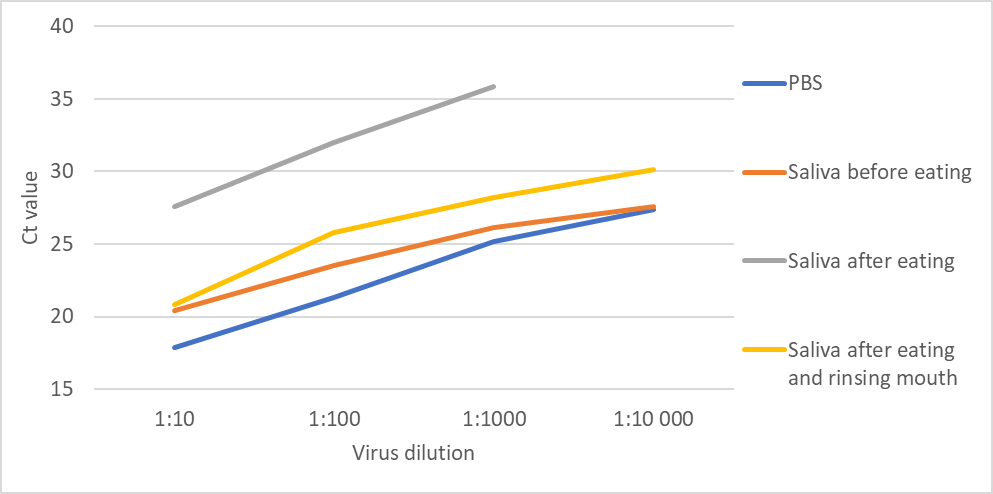


|  | Unspiked saliva | 1:10 | 1:100 | 1:1000 | 1:10 000 |
| --- | --- | --- | --- | --- | --- |
| PBS | neg | 17.89 | 21.34 | 25.165 | 27.38 |
| Saliva before eating | neg | 20.46 | 23.52 | 26.16 | 27.57 |
| Saliva after eating | neg | 27.61 | 31.98 | 35.83 | No Ct |
| Saliva after eating and rinsing mouth | neg | 20.84 | 25.82 | 28.22 | 30.17 |

**Fig. S8 Inhibitory effect of saliva in detection of SARS-CoV-2 RNA by PCR.** Saliva samples taken before eating, after eating, and after eating and rinsing mouth were spiked with a dilution series of Fin/20 strain RNA and tested with N Charité PCR. Figure was created with Microsoft Excel for Microsoft 365 MSO, version 2201^11^.

### Cleaning protocol in the hospital

### The COVID-19 ward and ICU were cleaned daily between 9 and 10 am and between 4 and 5 pm. The cleaning protocol included removal of secretion stains with a disinfectant (visible stains were removed also between cleaning rounds), waste and laundry removal, wash and rinse of dispenser buttons, taps, hand shower and sinks with a brush, wiping of all contact surfaces with damp cleaning cloth from top to bottom, and mopping the floor. Additionally, the patient spot was cleaned after the patient left the ward. With final cleaning the bed and mattress were carefully cleaned, and all bed clothes and curtains were changed. Cleaning protocols should avoid methods which generate aerosols.

### Supplementary references

1 IBM Documentation - SPSS Statistics 27.0.0. https://www.ibm.com/docs/en/spss-statistics/27.0.0.

2 Corman, V. M. *et al.* Detection of 2019 novel coronavirus (2019-nCoV) by real-time RT-PCR. *Euro Surveill* **25**, doi:10.2807/1560-7917.ES.2020.25.3.2000045 (2020).

3 CDC. *A CDC 2019-Novel Coronavirus (2019-nCoV) Real-Time RT-PCR Diagnostic Panel*, <<https://www.fda.gov/media/134922/download>> (2020).

4 Haveri, A. *et al.* Serological and molecular findings during SARS-CoV-2 infection: the first case study in Finland, January to February 2020. *Euro Surveill* **25**, doi:10.2807/1560-7917.ES.2020.25.11.2000266 (2020).

5 Chin, A. W. H. & Poon, L. L. M. Stability of SARS-CoV-2 in different environmental conditions - Authors' reply. *Lancet Microbe* **1**, e146, doi:10.1016/S2666-5247(20)30095-1 (2020).

6 CDC, C. *China CDC Primers and Probes for Detection 2019-nCoV*, <<http://ivdc.chinacdc.cn/kyjz/202001/t20200121_211337.html> > (2020).

7 Institut Pasteur, P. *Protocol: Real-Time RT-PCR Assays for the Detection of SARS-CoV-2*, <<https://www.who.int/docs/default-source/coronaviruse/real-time-rt-pcr-assays-for-the-detectionof-sars-cov-2-institut-pasteur-paris.pdf?sfvrsn=3662fcb6_2>> (2020).

8 Cantuti-Castelvetri, L. *et al.* Neuropilin-1 facilitates SARS-CoV-2 cell entry and infectivity. *Science* **370**, 856-860, doi:10.1126/science.abd2985 (2020).

9 Virtanen, J. *et al.* Kinetics of Neutralizing Antibodies of COVID-19 Patients Tested Using Clinical D614G, B.1.1.7, and B 1.351 Isolates in Microneutralization Assays. *Viruses* **13**, doi:10.3390/v13060996 (2021).

10 Etievant, S. *et al.* Performance Assessment of SARS-CoV-2 PCR Assays Developed by WHO Referral Laboratories. *J Clin Med* **9**, doi:10.3390/jcm9061871 (2020).

11 Microsoft Excel Spreadsheet Software ﻿https://www.microsoft.com/en-us/microsoft-365/excel
